# P77 Burden of MDR pathogens in hospitalized patients: a clinical microbiology study

**DOI:** 10.1093/jacamr/dlag102.083

**Published:** 2026-06-26

**Authors:** Greta Lila, Rezarta Bajrami, Yllka Begolli

**Affiliations:** Faculty of Medicine, University of Prishtina, Pristina, Kosovo; University Clinical Centre of Kosovo, Pristina, Kosovo; Faculty of Medicine, University of Prishtina, Pristina, Kosovo; National Institute of Public Health of Kosovo, Pristina, Kosovo; University Clinical Centre of Kosovo, Pristina, Kosovo

## Abstract

**Objectives:**

MDR organisms (MDROs) pose a growing threat in healthcare settings. Transmission and persistence of the resistant strain is determined by the availability of vulnerable patients, selective pressure exerted by antimicrobial use, increased potential for transmission from larger numbers of colonized or infected patients (‘colonization pressure’) and the impact of implementation and adherence to prevention efforts. This study investigated the prevalence, distribution, and microbiological profile of MDRO colonization/infection among hospitalized patients in University Clinical Center during the year 2025. Microbiological analysis targeted key MDROs, including *Acinetobacter baumannii*, *Klebsiella pneumoniae*, VRE, *Candida auris* and MRSA.

**Methods:**

Identification of all isolates was performed using MALDI-TOF MS (bioMérieux). Antimicrobial susceptibility testing was conducted using the VITEK^®^ 2 (bioMérieux) and BD-Phoenix automated system. Antimicrobial susceptibility results were interpreted according to EUCAST guidelines. The presence of genes coding for antibiotic resistance was determined using eazyplex^®^.

**Results:**

The overall MDROs prevalence for the investigated bacteria was 1002 (25.86%). The prevalence of MDR *A. baumannii* was 248 (24.75%). *Staphylococcus aureus* isolates were found in 145 samples and the prevalence of MRSA was 115 (79.3%). 20.86% of MRSA was in blood samples. *Enterococcus* spp. was isolated in138 (13.77%) samples and the prevalence of VRE was 68 (49.27%). 11.76% of VRE was found in blood samples. *C. auris* most frequently was isolated from urine samples 17 (8.76%). *K. pneumoniae* isolates was found in 277 (27.64%) of the samples and the prevalence of CRKP was found in 25(9.02%). CRKP most frequently was found in blood samples (8%) and urine samples (36%). A significant association was observed between blood samples and urine samples with the presence of MDROs. The most effective antibiotics were tigecycline and cefiderocol. In CRKP strains most frequently were detected the NDM and OXA-48 genes. We have found that 9 (36%) of the CRKP isolates were resistant to colistin, but no mcr-1 gene was detected. The majority of the MDRO samples were from ICUs.

**Conclusions:**

This study highlights the high prevalence and diverse spectrum of MDRO colonization/infection among different clinical specimens. Timely detection through active surveillance is essential for infection prevention and antimicrobial stewardship in our institution Additional prospective research is needed to assess the influence of comorbidities, duration of hospitalization, and previous antibiotic usage on the prevalence of MDROs in hospital settings.

